# Pigment Cell Differentiation in Sea Urchin Blastula-Derived Primary Cell Cultures

**DOI:** 10.3390/md12073874

**Published:** 2014-06-27

**Authors:** Natalya V. Ageenko, Konstantin V. Kiselev, Pavel S. Dmitrenok, Nelly A. Odintsova

**Affiliations:** 1 Cytotechnology Laboratory, A.V. Zhirmunsky Institute of Marine Biology, FEB RAS, Vladivostok 690041, Russia; 2 Far Eastern Federal University, Sukhanova Str. 8, Vladivostok 690950, Russia; E-Mail: nelodin54@yahoo.com; 3 Laboratory of Biotechnology, Institute of Biology and Soil Sciences, FEB RAS, Vladivostok 690022, Russia; E-Mail: kkv5@mail.ru; 4 Laboratory of Instrumental and Radioisotope Methods of Analysis, G.B. Elyakov Pacific Institute of Bioorganic Chemistry, FEB RAS, Vladivostok 690022, Russia; E-Mail: paveldmt@piboc.dvo.ru

**Keywords:** cell culture, echinochrome, gene expression, MALDI TOF MS, ESI MS, marine biotechnology, naphthoquinone pigments, pigment differentiation, proliferation, sea urchin

## Abstract

The quinone pigments of sea urchins, specifically echinochrome and spinochromes, are known for their effective antioxidant, antibacterial, antifungal, and antitumor activities. We developed *in vitro* technology for inducing pigment differentiation in cell culture. The intensification of the pigment differentiation was accompanied by a simultaneous decrease in cell proliferation. The number of pigment cells was two-fold higher in the cells cultivated in the coelomic fluids of injured sea urchins than in those intact. The possible roles of the specific components of the coelomic fluids in the pigment differentiation process and the quantitative measurement of the production of naphthoquinone pigments during cultivation were examined by MALDI and electrospray ionization mass spectrometry. Echinochrome A and spinochrome E were produced by the cultivated cells of the sand dollar *Scaphechinus mirabilis* in all tested media, while only spinochromes were found in the cultivated cells of another sea urchin, *Strongylocentrotus intermedius*. The expression of genes associated with the induction of pigment differentiation was increased in cells cultivated in the presence of shikimic acid, a precursor of naphthoquinone pigments. Our results should contribute to the development of new techniques in marine biotechnology, including the generation of cell cultures producing complex bioactive compounds with therapeutic potential.

## 1. Introduction

**Figure 1:**
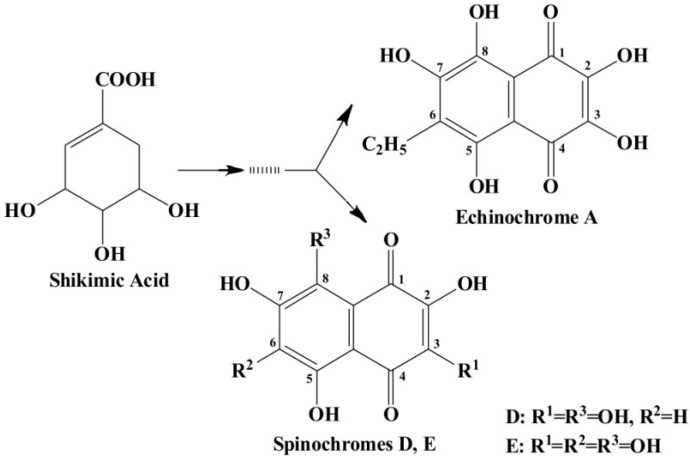
The structures of shikimic acid, a precursor of naphthoquinone pigments, and echinochrome and spinochromes in accordance with a previous report [7].

Marine inhabitants are the most phylogenetically diverse organisms, demonstrating a significant potential for biodiscovery research [1] and as possible sources of valuable biologically active substances for the pharmaceutical and food industries [2]. For example, sea urchins are a source of pharmacologically important quinone pigments—specifically echinochrome and the spinochromes—that constitute a group of polyketide compounds. Like many marine secondary metabolites, polyketide compounds are known for their highly effective antioxidant, antibacterial, antifungal, and antitumor activities. In addition, these compounds may play decisive roles in the regulation of lipid peroxidation and in immune defense [3,4,5,6]. They are generated via a series of enzymatic, oxidative and photochemical reactions from shikimic acid (ShA)—a precursor of naphthoquinone pigments (Figure 1) [7]. Echinochrome is one of these pigments and is synthesized in the sea urchin pigment cells demonstrating a strong bactericidal effect during embryonic and larval development [8,9]. The morphology and behavior of pigment cells are similar to those of macrophages, confirming the involvement of sea urchin pigment cells in the larval immune system [10]. A drug (Histochrome^®^, Moscow, Russia) with cardiological and ophthalmological activity based on the echinochrome structure has been developed to correct metabolic processes and act as an oxygen transporter [11].

An industrial-scale procurement of echinochrome could lead to the extinction of the organisms that produce this substance. Therefore, cultured sea urchin pigment cells might provide a source of pharmacologically important quinone pigments, as well as help reduce the impact on the adult sea urchin population and pave the way for solving several pharmacological problems.

The aim of our work is to develop a technology for directed-pigment differentiation in sea urchin culture for solving practical tasks in marine biotechnology. Two sea urchin species differing in their number of embryonic pigment cells are chosen: the sea urchin *Strongylocentrotus intermedius* and the sand dollar *Scaphechinus mirabilis.* As shown in [12], the embryos of the sand dollar contain a lot of pigment cells. Previously, Calestani *et al.* [13] revealed three groups of genes expressed in sea urchin pigment cells that play key roles in the biosynthesis of naphthoquinone pigments [14]. The central parts of these genes in *S. intermedius* have been reported to be similar to those of the same genes in the closely related sea urchin *S. purpuratus* [15]. In this study, we evaluated the gene expression associated with the induction of pigment differentiation in cell cultures.

As previously shown, sand dollar embryos transfected with the foreign gene (the yeast *gal4* gene encoding the transcription activator) were dissociated into single cells that produced pigments after one month of cultivation [16]. We then developed an *in vitro* technology for inducing pigment differentiation without transfecting foreign genes into the sea urchin embryos but using the coelomic fluids from sea urchins [15]. Here, to estimate the contributions of the specific components of the coelomic fluids from sea urchins to the pigment differentiation process and the production of naphthoquinone pigments during cultivation, we used matrix assisted laser desorption/ionization (MALDI) time-of-flight (TOF) mass spectrometry and electrospray ionization (ESI) mass spectrometry.

This study has three main results. First, we developed an *in vitro* technology for inducing pigment differentiation in cell culture. Second, our data support the hypothesis that specific components of sea urchin coelomic fluids might act as inductive signals in pigment differentiation through the regulation of genes implicated in naphthoquinone synthesis. Third, echinochrome was produced only in the sand dollar cells, and its maximum level was found in cells cultured in coelomic fluids rather than seawater.

## 2. Results and Discussion

### 2.1. Differentiation of Pigment Cells in a Blastula-Derived Cell Culture

**Figure 2:**
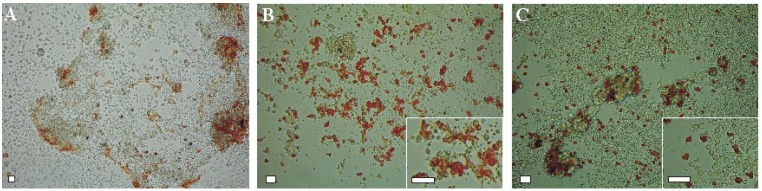
Embryonic pigment cells in a blastula-derived cell culture of the sea urchin *Scaphechinus mirabilis* cultivated for 3 days. The cells were cultivated in seawater (**A**); the coelomic fluid of intact sea urchins (**B**); or the coelomic fluid of injured sea urchins (**C**). All culture media were supplemented with 2% fetal bovine serum. Insets in **B** and **C**: higher magnifications. Note the change in morphology of the pigment cells depending on the medium tested. Scale bar, 10 μm.

**Figure 3:**
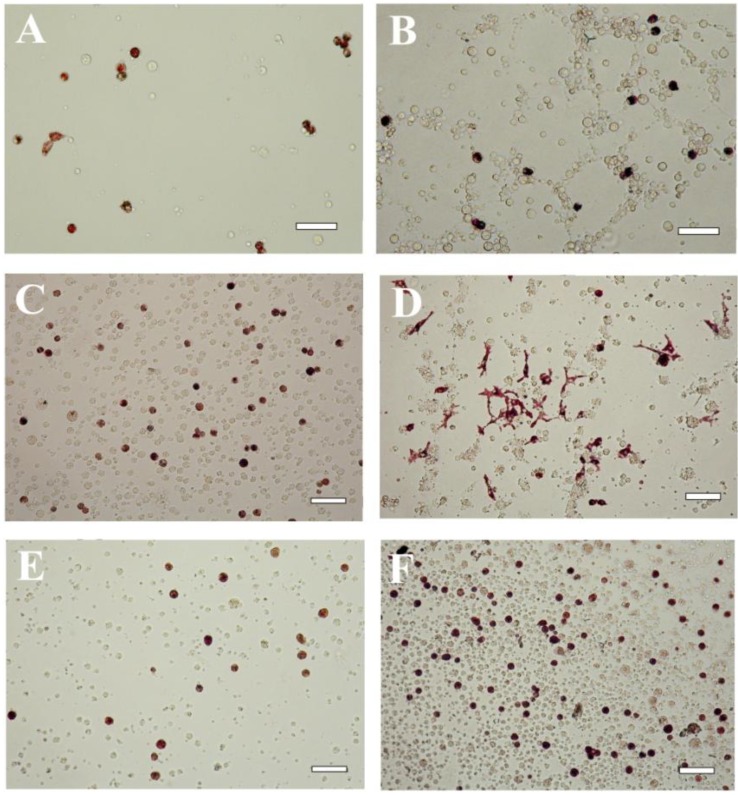
Embryonic pigment cells in a blastula-derived cell culture of the sea urchin *Strongylocentrotus intermedius* cultivated for 5 (**A**,**B**); 17 (**C**,**D**) or 41 days (**E**,**F**). **A**, **C**, **E**—cells cultivated in the coelomic fluid of intact sea urchins; **B**, **D**, **F**—cells cultivated in the coelomic fluid of injured sea urchins. The coelomic fluid was supplemented with 2% fetal bovine serum. Note the change in morphology of the pigment cells during cultivation. Scale bar, 10 μm.

The growth patterns and morphology of cultured cells is often determined by peculiarities of the culture medium. To evaluate the effect of different culture media on the development of pigment differentiation in the cell cultures of both sea urchin species, we tested three types of media (Figure 2 and Figure 3): seawater, the coelomic fluid preparations of control sea urchins and injured sea urchins. Injured sea urchins were obtained by needle pricks in the area of Aristotle’s lantern (see the Experimental Section). The first distinctions in the appearance of pigment cells and their pigmentation became obvious after 3–7 days of cultivation. The cells cultivated in seawater were faintly pigmented and not numerous, whereas the pigment cells were more abundant in the coelomic fluid-cultivated cells. This picture was observed both in a short-time culture of the sea urchin *S. mirabilis* and in a long-time culture of the sea urchin *S. intermedius*.

If the coelomic fluid of intact sea urchins was used as the medium, the pigment cells of *S. mirabilis* were well attached and spread during three days in culture (Figure 2B, insert). However, when the injured sea urchin coelomic fluid was used, most of the pigment cells were rounded and failed to spread (Figure 2C, insert).

**Figure 4:**
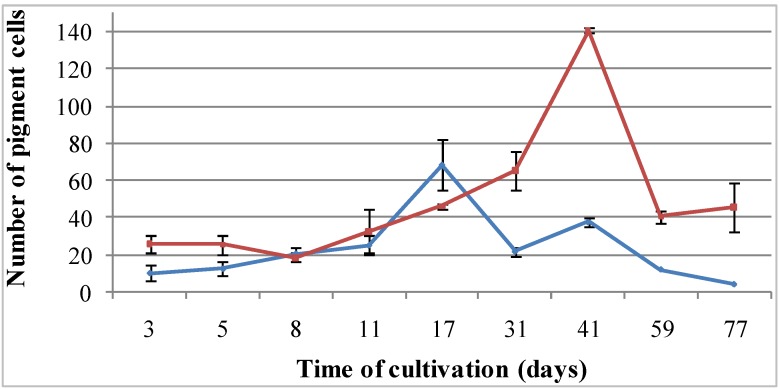
Cellular dynamics of pigment cells cultivated in coelomic fluids of intact (**blue** line) and injured (**red** line) sea urchins for 2.5 months. At least 500 cells were counted in each tested culture medium.

The medium-dependent differences in cell morphology were also seen in the sea urchin *S. intermedius*. The pigment differentiation of the cells in the seawater occurred slowly and less extensively (data not shown) than that of the cells in the coelomic fluids. Furthermore, the pigment cells in this species changed their morphology during prolonged cultivation for 20 days in the same medium (Figure 3A–D). When the cells were cultivated in injured sea urchin coelomic fluid, the maximal spreading of the pigment cells was observed on Day 17 of cultivation (Figure 3D). Upon further culture, the cells became rounded (Figure 3E,F) and increased in number up to Day 41 of cultivation (Figure 4). Signs of cell degeneration were still detectable after two months of culturing.

The maximal number of pigmented cells of *S. intermedius* was detected on Day 17, when the cells were cultivated in the coelomic fluid of intact sea urchins (Figure 4, blue line). When the cells were cultivated in the injured sea urchin coelomic fluid, that maximal number was recorded on Day 41 (Figure 4, red line).

The number of pigment cells was dependent on the coelomic fluid used: twice as many pigment cells were detected in the cells cultivated in the injured sea urchin coelomic fluid as in that from intact sea urchins*.* In contrast*,* the number of pigment cells was significantly lower among cells cultured in in seawater (data not shown).

### 2.2. Cell Proliferation in Culture

**Figure 5:**
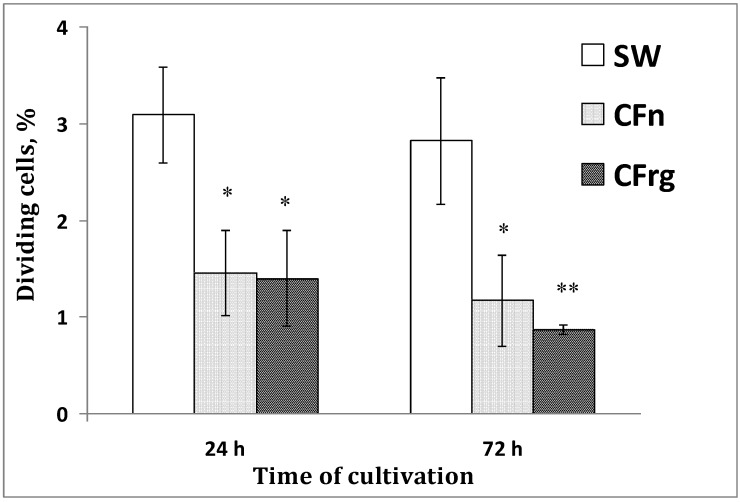
The number of dividing cells in a blastula-derived culture of the sea urchin *S. intermedius* depending upon culture medium tested. The cells were cultivated for 24–72 h. The proportion of phospho-H3-histone-positive cells among the total number of examined cells (determined by DAPI staining of nuclei) was counted. SW—seawater; CFn—coelomic fluid obtained from intact sea urchins; CFreg—coelomic fluid obtained from injured sea urchins. * *p* < 0.05; ** *p* < 0.01 *versus* values of dividing cells cultivated in the sea water (24 h).

It is well known that cell proliferation and differentiation are interrelated processes. To compare the contribution of these processes—pigment differentiation (as described above) and cell proliferation—in sea urchin culture, we detected dividing cells with antibodies to phospho-H3-histone, a well-known cell proliferation marker for many organisms [17,18,19]. At least 300–500 DAPI-stained cells were examined for each experiment with the culture media tested, and the proportion of cells positive for phospho-H3-histone relative to the total number of cells was determined. The number of dividing cells was higher in cells cultured in seawater than in those cultured in the coelomic fluids, where cell division was weak (Figure 5).

A significant difference in the number of dividing cells in the different media after one day of cultivation was found, and this difference increased for three days, when a general decrease in mitotic activity was then detected. In most cases, the cultures survived well, but their proliferation rate was low.

**Figure 6:**
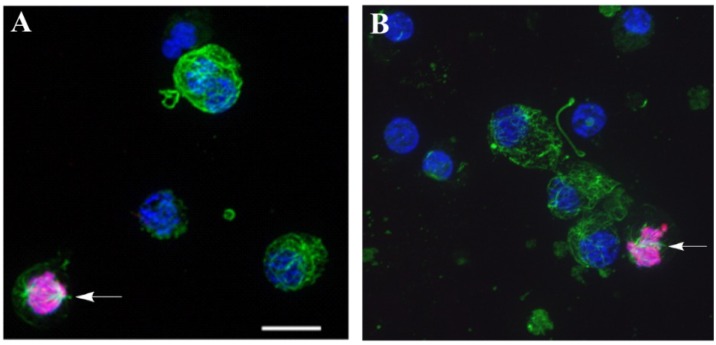
Immunofluorescence detection of dividing (phospho-H3-histone-positive) cells in a blastula-derived culture. The preparations were imaged via confocal microscopy. The cells were cultivated on fibronectin-coated coverslips in coelomic fluid obtained from injured sea urchins (**A**) and in seawater (**B**) for 12 h and then labeled with Abs against phospho-H3-histone Abs for the detection of dividing cells (**red**) and tubulin (**green**) for the detection of microtubules. The nuclei were stained with DAPI (**blue**). Arrows show ph-H3-histone-positive cells. Scale bar, 10 µm.

Dividing (phospho-H3-histone+) cells were detected from 6 to 72 h in all media tested, but the maximal number of dividing cells was found in seawater. The decrease in cell proliferation in the coelomic fluids was accompanied by a simultaneous intensification of the pigment cell differentiation. As already noted, the composition of the culture medium affected the rate of appearance of the pigment cells during cultivation. Figure 6A,B arrows, shows dividing cells in both of the coelomic fluid and seawater after 12 h of cultivation (see details of the immunocytochemical manipulations in the Experimental Section).

### 2.3. MALDI MS Analysis of Coelomic Fluids Obtained from Intact and Injured Sea Urchins

**Figure 7:**
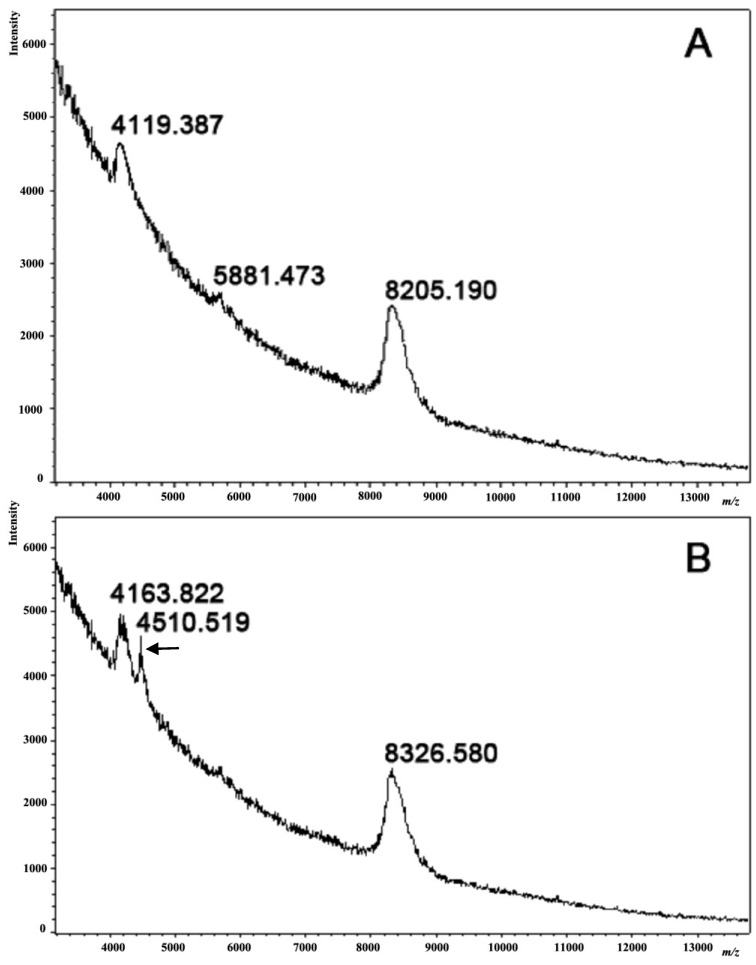
The mass spectra of coelomic fluids obtained from intact (**A**) and injured (**B**) sea urchins by MALDI TOF MS using a mass spectrometer (Ultraflex-III TOF/TOF, Bruker Daltonics, Germany). Note a new peak in the coelomic fluid of the injured sea urchins, corresponding to a protein with MW near 4500 Da (marked by an arrow), as well as the shift of the basic components of the intact coelomic fluid to approximately 40–120 Da. The coelomic fluids from three independent experiments were analyzed.

To estimate the contribution of specific components in intact and injured sea urchin coelomic fluids to the cell pigment differentiation process during cultivation and to understand its origin, we conducted MALDI MS analyses of both coelomic fluids of *S. intermedius*. Figure 7 illustrates the MALDI mass spectra obtained for proteins with a molecular mass of 4000–9000 Da. Two well-defined peaks corresponding to proteins of 8205 Da and 4119 Da were detected in the spectrum of the intact sea urchin coelomic fluid (Figure 7A). The spectrum of coelomic fluid obtained from the injured sea urchins exhibited three peaks: 8327 Da, 4511 Da, and 4164 Da (Figure 7B). A comparison of the coelomic fluid profiles revealed a new peak in the coelomic fluid of the injured sea urchins corresponding to a protein with a molecular weight of around 4500 Da and a shift of approximately 40–120 Da from the basic components in the intact sea urchin coelomic fluid, which may be due to a protein modification such as phosphorylation, sulfation, *etc.* The molecular weights of the proteins were measured using MALDI MS with standard precision (0.1%) based on the external calibration programs included (Protein Calibration Mixture II, Bruker Daltonik, Germany). The new peak in the spectrum (4511 Da) indicates a change in the composition of the proteins in the coelomic fluid after injury in comparison to the norm. Although we could not identify the molecular nature of the coelomic fluid proteins, our data clearly demonstrated a qualitative difference in the composition of normal coelomic-fluid proteins and that of coelomic-fluid proteins following injury.

### 2.4. Naphthoquinone Pigment Production in Cultivated Sea Urchin Cells

**Figure 8:**
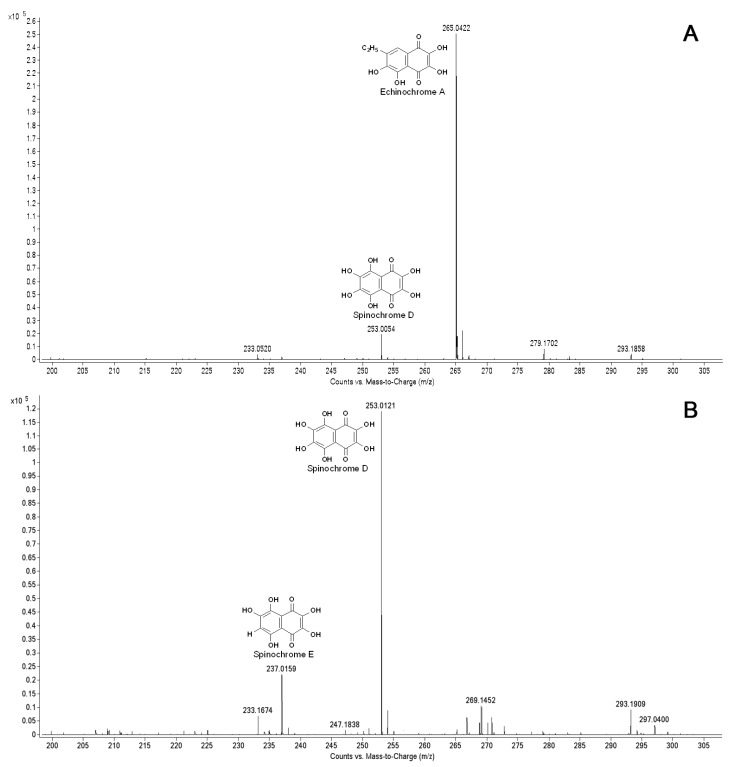
ESI MS profiles of naphthoquinone pigments from sea urchin cell cultures: A—the pigment extracts from the cells of the sea urchin *S. mirabilis* after 3-day cultivation in seawater; B—the pigment extracts from the cells of the sea urchin *S. intermedius* after 6-day cultivation in seawater.

To elucidate the naphthoquinone pigment profile in the cultivated sea urchin cells and the production of pigments during cultivation, we performed ESI MS. As shown in Figure 8A, a significant peak of echinochrome A and a small peak corresponding to spinochrome E were detected in the pigment extracts from the sand dollar *S. mirabilis*, while only the peaks corresponding to spinochromes D and E were recorded in the pigment cell extracts from *S. intermedius* (Figure 8B). Thus, a distinct difference in pigment expression was demonstrated in the cells of the two sea urchin species.

The results of a semi-quantitative measurement (using ESI MS) of the echinochrome and spinochrome production in cells cultivated in the different media are presented in Table 1. Echinochrome A was produced by cultivated *S. mirabilis* cells in all of the tested media, and it was well defined in all of the spectra. The highest level of echinochrome A was detected in the cells cultured in the coelomic fluids as compared to those cultured in seawater. In contrast, no significant differences were seen in the spinochrome D and E levels in the tested media for the cultivated cells of the other sea urchin, *S. intermedius* (Table 1).

**Table 1:** Naphthoquinone pigment production in sea urchin cultivated cells. Data are presented as the mean ± standard error from two independent experiments (ESI MS). SW—seawater; CFn—coelomic fluid obtained from intact sea urchins; CFreg—coelomic fluid obtained from injured sea urchins.

Pigments, mg/g of Fresh Biomass of Cells	M.W.	Cell Culture Medium		
		SW	CFn	CFreg
*Scaphechinus mirabilis*				
*Spinochrome E* 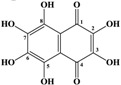	254.00	0.021 ± 0.003	0.062 ± 0.007	0.054 ± 0.006
*Echinochrome A* 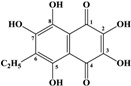	266.04	0.250 ± 0.026	0.640± 0.061	0.540 ± 0.055
*Strongylocentrotus intermedius*				
*Spinochrome E* 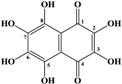	254.00	0.024 ± 0.004	0.013 ± 0.002	0.015 ± 0.002
*Spinochrome D* 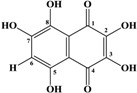	238.02	0.052 ± 0.006	0.044 ± 0.005	0.062 ± 0.007

### 2.5. Expression of the pks Genes in Cultivated Sea Urchin Cells. Experiments with Shikimic Acid, a Precursor of Naphthoquinone Pigments

**Figure 9:**
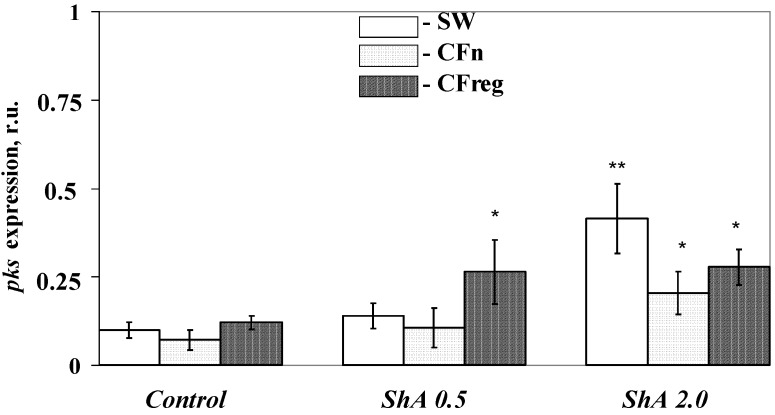
Effect of Shikimic Acid (ShA) on the *pks* gene expression in a blastula-derived cell culture of the sea urchin *Strongylocentrotus intermedius.* ShA concentrations tested: 0.5 mM and 2.0 mM. *Y*-axis—relative units (r.u.). The cells were cultivated with ShA for 4 days in: seawater (SW), coelomic fluid of intact sea urchins (CFn), and coelomic fluid of injured sea urchins (CFreg). After this period, total RNA was isolated from cultivated cells for the following real-time PCR. Сontrol cells are the cells cultivated without ShA. * *p* < 0.05; ** *p* < 0.01 *versus* values of *pks* expression in the appropriate control conditions.

To understand the molecular mechanisms underlying sea urchin pigment specialization in culture, we evaluated the gene expression associated with the induction of pigment differentiation, particularly the *pks* genes. The expression level of these genes (estimated by quantitative real-time PCR) in sea urchin cells cultivated in different culture media is presented in relative units in Figure 9 (see the Experimental Section). The gene expression in sea urchin cells cultivated in the coelomic fluid of injured sea urchins increased more than 1.2-fold in comparison to the cells cultivated in seawater, whereas the *pks* expression in the cells cultivated in the coelomic fluid from intact sea urchins decreased by almost 1.4-fold; however, these differences were insignificant. The addition of a high concentration of ShА, a precursor of naphthoquinone pigments, (2 mM) to the sea urchin cells resulted in a marked intensification of the gene expression in all media tested (2.3–4.2-fold) compared to the control cells (Figure 9), but the effect was especially significant in seawater. In contrast, adding ShА of a lower concentration (0.5 mM) increased *pks* expression only in the cells cultivated in injured sea urchin coelomic fluid (2.2-fold). No apparent effect on *pks* expression was detected in the cells cultivated with 0.5 mM ShA in seawater or intact sea urchin coelomic fluid. These data can be explained by the presence of some specific components in the sea urchin coelomic fluids. The marked intensification of the *pks* expression (2.2-fold) in the presence of 0.5 mM ShА only in the cells cultivated in injured sea urchin coelomic fluid is additional evidence of a cellular reaction in response to injury signals. Our findings showing a significant difference between the number of sea urchin pigment cells cultivated in the coelomic fluids of injured and intact sea urchins support this suggestion.

### 2.6. Discussion

Marine biotechnology progress is associated with the search, characterization, and development of optimal methods for obtaining new substances from marine organisms with pharmaceutically interesting biological activity, and many are of enormous scientific interest. On the one hand, marine organisms are a source of unique marine secondary metabolites that are the basis for developing new and improved natural products for commercial purposes [2]. On the other hand, they include a variety of phylogenetic groups that are important for understanding the evolutionary history of life. Some, such as the echinoderms, are members of a phylum of deuterostomate metazoans and occupy a key position in the early steps in chordate evolution [20,21]. As shown by Evans-Illidge *et al.* [1], the ancestral early-metazoans (Porifera), early eumetazoans (Cnidaria) and some deuterostomes (Echinodermata) have a very high concentration of bioactive compounds.

Here, we have shown that the culture medium composition affects the rate of appearance of pigment cells. In seawater, the number of dividing cells was higher than when the cells were cultured in the coelomic fluids, but the pigment cell differentiation occurred slowly and less extensively. An increase in cell pigmentation in the coelomic fluids probably leads to a decrease in the number of dividing cells in comparison to the cells incubated in seawater. In the early stages of cultivation, we detected dividing cells and a few pigment cells, but later during the pigment cell differentiation, we observed a decrease in the number of dividing cells and a corresponding increase in the number of pigment cells. This is not surprising: either proliferation or differentiation usually dominates in cell cultures. A significant decrease in the number of dividing cells can be explained by a general reduction of mitotic activity during cultivation that has been previously reported for many invertebrate cells after 7–10 days of cultivation [22,23].

We proposed that a significant difference in the proportion of pigment cells in the cultures grown in the coelomic fluids of intact and injured sea urchins in comparison to those cultivated in seawater was connected to the specific components of coelomic fluids that increase the adhesion of cultivated cells. Recently, approximately 30 proteins that may be involved in adhesion were uncovered by a large-scale proteomic analysis of sea urchin coelomic fluid [24]. Thus, far, a proteomic analysis has only been performed on normal sea urchin coelomic fluid.

In this study, we analyzed the coelomic fluid of both normal and injured sea urchins, and detected qualitative differences in their composition. Our results from the overview of integral MALDI mass spectra of sea urchin coelomic fluids have shown that new peaks appear in the coelomic fluid obtained from injured sea urchins. These new peaks may correspond to stress proteins produced after injury. As recently reported, relatively abundant proteins involved in stress responses were identified in the coelomic fluid of the sea urchin *S. purpuratus* [24]. Homologues of these proteins have been found throughout the animal kingdom and are often used as markers for environmental stress [25]. We have suggested that the new coelomic fluid proteins from the injured sea urchins may be related to stress-response proteins. The MALDI MS data together with the results for *pks* gene expression suggest that the specific components of sea urchin coelomic fluids play a role in pigment differentiation through the regulation of the genes implicated in naphthoquinone synthesis. Analyzing cells cultivated in the presence of 0.5 mM ShА, we showed that *pks* gene expression was two-fold higher in cells cultivated in the injured sea urchin coelomic fluid relative to cells cultivated in that from intact sea urchins. A higher proportion of cells containing naphthoquinones in response to different conditions of stress could be a consequence of a protective reaction and be important as a defense mechanism [26,27].

We found that echinochrome was produced only in the sand dollar cells and its content in cells grown in coelomic fluid was higher than that in cells grown in seawater. This finding suggests the existence of a specific regulatory factor that may induce pigmentation activity and subsequent naphthoquinone production in these coelomic fluids. Our morphological observations of the cultured cells correlated with the semi-quantitative data obtained using ESI MS. Unfortunately, not all of the experiments could be performed on the *S. mirabilis* cell cultures due to the difficulty in obtaining the sand dollar cells free of bacterial contamination.

## 3. Experimental Section

### 3.1. Animals

Adult sea urchins (*S. intermedius* and *S. mirabilis*, Echinoidae (Agassiz, 1863)), were collected during the breeding season (Vostok Bay, the Sea of Japan), and they were kept in aquaria filled with running, aerated seawater at 16 °C. Before experiments were conducted, the animals were rinsed free of any debris with UV-sterilized filtered seawater. Experiments were performed at the Marine Biological Station «Vostok» (A.V. Zhirmunsky Institute of Marine Biology, Vladivostok, Russia FEB RAS). Gametes and embryos were obtained by the KCl injection method as described before [15]. The embryonic material was placed in tanks with UV-sterilized seawater (18 °C) throughout development and harvested at the mesenchymal blastula (16 h post fertilization, hpf).

### 3.2. Cell Culture

Developing sea urchin embryos were collected on a fine 30-μm nylon mesh and dissociated into single cells with 0.25% collagenase at 17 °C (for 20–30 min) as described earlier [15]. The resulting cell suspension containing all cell types was washed several times in seawater with antibiotics, and then sterile seawater supplemented with 2% fetal calf serum, FCS (Sigma, St. Louis, MO, USA) was added. Cell viability was estimated at different time points by a trypan blue exclusion test. The cells were seeded at the density of 6 × 10^6^–8 × 10^6^ cells/mL in plastic Petri dishes (Lux Culture Dishes, MP Biomedicals, Santa Ana, CA, USA), and after two days of cultivation, a subset of cells (after several strokes of gentle pipetting) at the density of 3 × 10^6^–4 × 10^6^ cells/mL was transferred into new plastic Petri dishes (Nunc, Nunclon Surface, Roskilde, Denmark) on the coverslips for further culturing. We used three types of the cell culture media (98%) supplemented with 2% FCS: seawater and the coelomic fluid preparations of intact and injured sea urchins (*S. intermedius*) that were obtained as described in [15]. In few, a day after damaging sea urchins, the coelomic fluids from control and injured sea urchins were collected by puncture in the area of Aristotle’s lantern. After removal of the clotting of coelomocytes by centrifugation, the supernatant was sterilized by filtration (0.22 μm, Millipore, CA, USA). The cell cultures were maintained by replacement of 50% of the medium at 3–5-day intervals for 3–42 days at 17 °C. To observe primary cell cultures during the overall period we used an inverted microscope Axiovert 200M (Carl Zeiss, Jena, Germany).

### 3.3. Immunohistochemical Analysis

After cultivation on fibronectin-coated or non-coated coverslips for 12–72 h, the cells were fixed in 4% paraformaldehyde (Sigma) in PBS, pH 7.8, for 7–10 min at 4 °C and rinsed three times in cold PBS. The material was stored in PBS with 0.03% NaN_3_ at 4 °C until needed. To reduce non-specific binding, the samples were incubated overnight in a blocking solution containing 10% normal goat serum (Sigma), 0.25% BSA, 0.1% Triton X-100, and 0.03% NaN_3_ in PBS. The cells were first incubated for 8 h at 10 °C in blocking solution with the following primary antibodies (Abs): mouse monoclonal anti-α acetylated tubulin (Sigma, diluted 1:1000) and rabbit polyclonal anti-phospho-H3 histone (Merck, Millipore, Germany, diluted 1:500). As secondary Abs, we used goat anti-mouse (GAM) or goat anti-rabbit (GAR) secondary Abs conjugated to Alexa Fluor 488, 546 or 633 (Molecular Probes, Eugene, OR, USA) at a dilution of 1:1000–1:2000 in PBS for 2 h at RT. The specimens were then washed and embedded in the Vectashield mounting medium (Vector Laboratories, Burlingame, CA, USA) containing 0.1 μg/mL DAPI to reveal the nuclei. For negative controls, primary Abs were omitted from the staining protocol. Specimens were analyzed with a confocal laser scanning microscope (Zeiss LSM 780) equipped with a high sensitivity GaAsP detector (with the 63× or 100× oil-immersion objectives). For 3D reconstructions, ImageJ software (NIH) was used. The number of dividing cells in each type of culture medium was estimated by calculating the percentage of phospho-H3 histone-positive cells among the total number of examined cells (determined by DAPI staining of nuclei). At least 300–500 DAPI-stained cells were counted in each experiment for each type of culture medium tested.

### 3.4. MALDI and ESI MS

Coelomic fluids obtained from intact and injured sea urchins, as described previously [15] were selected for MALDI-MS analysis. The mass spectra were registered using an Ultraflex-III TOF/TOF mass spectrometer (Bruker Daltonics, Bremen, Germany), equipped with a smart-beam laser (355 nm) having a pulse frequency of 100 Hz and a pulse width of 3 ns (PIBOC, FEB RAS). The mass spectrometer was used in the linear mode with delayed ion extraction and with the matrix alpha-cyano-4-hydroxy-cinnamic acid (Bruker Daltonics). The Protein Calibration Standard I (Bruker Daltonics) (1 μL) was added as the internal standard.

Metabolites were extracted from cultivated sea urchin cells. Quinone pigments were extracted with 1 mL of acidified ethanol (1 part 25% HCl and 3 parts of 96% ethanol) for 24 h at 4 °C, as described [28]. Diethyl ether was added to supernatants, and an organic layer containing quinones was washed with 5% NaCl until the acid was almost removed. The ether solution was dried over anhydrous sodium sulfate. The extract including the pigments was stored at −25 °C in the dark, and it was then analyzed by ESI-MS using a mass spectrometer Agilent 6510 Q-TOF LC/MS (Agilent Technologies, Santa Clara, CA, USA).

The alcoholic extracts were obtained from cultivated cells (5 × 10^6^ cells/mL) after 3 and 6 days in culture. The sample solution was membrane-filtered (0.45 μm, Millipore), and 2 μL aliquots were used for analysis.

The mass spectra were recorded in the negative-ion detection mode within the *m/z* mass range of 100–800 and at mass-to-charge ratio (*m/z*) of 70–500 for MS/MS spectra (scan time 1 s). The quinonoid pigments were identified by exact mass measurement of deprotonated molecula [M − H]^−^ in the MS spectra and by comparisons of the characteristic MS/MS spectra with the MS/MS spectra of pure naphthoquinone standards. The MS/MS spectra were recorded in the MS/MS mode using a collision energy ranging from 20 V to 35 V; the precursor ions were isolated with an isolation width of 1.3 *m/z*. The mass spectrometer was calibrated using the ESI-L Low Concentration Tuning Mix (Agilent Technologies, USA). A high-resolution MS analysis was performed by adding the Reference Mix (Agilent Technologies, USA) through a reference sprayer in a Dual-ESI source. The data were analyzed with FlexAnalysis Version 3.0 (Bruker Daltonics, Bremen, Germany).

### 3.5. Quantitative Real-Time PCR (Q-Real-Time PCR)

Sterile solutions of ShA (Sigma) in seawater at the desired concentrations (0.5 mM and 2.0 mM) were added in a blastula-derived cell culture of the sea urchin *S. intermedius* after seeding, and the cells were incubated during four days. After this period, total RNAs from cultivated sea urchin cells were extracted with Yellow Solve reagent (Clonogen, St. Petersburg, Russia) and treated with DNase I (Sileks, Moscow, Russia) to remove genomic DNA. The first strand of cDNA was synthesized using 1.5 μg of total RNA as a template with the Reverse Transcription System (Sileks) in a 50 μL reaction volume as described previously [5]. The 0.5–2 μL samples of reverse transcription products were then amplified by PCR on the sea urchin actin gene using the primers 5′CAA CGG ATC CGG TAT GGT GAA GGC and 5′TCC AGA CGG AGG ATG GCG TGG GGA. The PCR reactions were made using iCycler thermocycler (Bio-Rad Laboratories, Inc., Hercules, CA, USA) with the conditions: one cycle of 2 min at 95 °C followed by 40 cycles of 15 s at 95 °C, 15 s at 50 °C and 35 s at 72 °C with a final extension cycle of 10 min at 72 °C. In the following Q-real-time PCR analyses, we used only those reverse transcription reactions that resulted in 500 bp PCR products for the *actin* gene. We discarded those reverse transcription reactions that resulted in both 500 and 700 bp PCR products for the *actin* gene, which indicated DNA contamination.

Quantitative real-time PCR was performed using the established protocol [15]. For TaqMan Q-real-time RT-PCR, cDNAs were amplified in 20 μL of the reaction mixture containing 1× TaqMan Buffer, 2.5 mM MgCl_2_, 250 μM of each deoxynucleotide, 1 U Taq DNA polymerase, 0.5–2 μL cDNA samples, and 0.25 μM of each primer and probe (Real-Time PCR Kit, Syntol, Moscow, Russia). The amplification conditions consisted of one cycle of 2 min at 95 °C followed by 50 cycles of 10 s at 95 °C and 25 s at 62 °C. The TaqMan PCR assays were performed in an iCycler thermocycler supplied with the iQ5 Multicolor Real-Time PCR detection system (Bio-Rad Laboratories, Inc., Hercules, CA, USA) and data were analyzed with the iQ5 Optical System Software v.2.0 as described previously [6] and presented in relative units. The *S. intermedius actin* gene (GenBank accession number DQ229162) and *ubiquitin* gene (LOC754856) were used as an endogenous control to normalize variance in the quality and the amount of cDNA used in each Q-real-time RT-PCR experiment. The data were summarized from five independent experiments. Primers and TaqMan probes for the *actin*, *ubiquitin*, *pks* genes used in Q-real-time PCR were described previously [6,15].

### 3.6. Statistical Analysis

The data are presented as mean ± standard error of the mean (SE). Data from the experiments were subjected to a one-way analysis of variance (ANOVA) with Tukey’s pairwise comparison test at *p* = 0.05 with the use of the Microsoft Office Excel 2003 program.

## 4. Conclusions

This study provides evidence for the production of naphthoquinone pigments in cultivated sea urchin cells. Echinochrome A and spinochrome E were produced by cultivated cells from the sand dollar *S. mirabilis* in all tested media, but only spinochromes were recorded in the cultivated cells from the sea urchin *S. intermedius*. Our data support the hypothesis that specific components of sea urchin coelomic fluids might act as inductive signals in pigment differentiation. The differentiation of pigment cells is accompanied by the active expression of genes involved in naphthoquinone synthesis and appears to be important for defense processes. Thus, our findings and the technology developed for directed pigment cell differentiation in culture may be instrumental in solving some practical tasks in marine biotechnology, including the generation of cell cultures producing complex bioactive compounds with therapeutic potential.
